# Outcomes of Stenting in Atypical Idiopathic Intracranial Hypertension Patients With Transverse Sinus Stenosis

**DOI:** 10.1002/brb3.70351

**Published:** 2025-02-19

**Authors:** Shuang Song, Zhongao Guan, Shuran Wang, Yawen Gan, Fangguang Chen, Jie He, Ketao Tu, Zhongrong Miao, Xu Tong, Dapeng Mo, Ke Pang

**Affiliations:** ^1^ Department of Interventional Neuroradiology, Beijing Tiantan Hospital Capital Medical University Beijing China; ^2^ Department of Neurology the Second Hospital of Hebei Medical University Shijiazhuang Hebei China; ^3^ Department of Ophthalmology, Beijing Tiantan Hospital Capital Medical University Beijing China

**Keywords:** idiopathic intracranial hypertension, transverse sinus stenosis, venous sinus stenting

## Abstract

**Background:**

Venous sinus stenting (VSS) has emerged as a novel treatment option for idiopathic intracranial hypertension (IIH) with transverse sinus stenosis (TSS). However, the efficacy of stenting in atypical IIH patients (body mass index [BMI] < 24 kg/m^2^, ≥ 50 years of age, or male) has not been fully investigated.

**Methods:**

The data of IIH + TSS patients who experienced conventional medical treatment failure and received VSS treatment at Beijing Tiantan Hospital in China between May 2021 and December 2022 were retrospectively reviewed in a prospectively maintained database. Baseline characteristics and periprocedural features were collected, and follow‐up clinical outcomes (intracranial pressure [ICP], stent‐adjacent stenosis, and symptoms and ophthalmic improvements) were compared between typical and atypical patients.

**Results:**

Data were available for 24 IIH + VSS patients. The median age was 37 years, and 95.8% were female. There were 9/24 atypical patients (37.5%). At baseline, atypical patients had milder visual field defects than typical patients did, as reflected by the mean deviation (MD) of the worse eye (median of −3.08 decibel (dB) vs. −7.63 dB, *p* = 0.027). Periprocedurally, 1–2 carotid self‐expandable stents with 7–9 mm in length and 40–50 mm in diameter were deployed at the site of the TSS. The median follow‐up duration was 12 months (range 8–22 months). At the last visit of follow‐up, stenting intervention significantly decreased the ICP and grade of papilledema and improved the best corrected visual acuity in the worse eye and MD in the fellow eye in both typical and atypical patients. The clinical outcomes of typical and atypical IIH + TSS patients were not significantly different, except that the MD of the worse eye in the atypical group was greater than that in the typical group (−1.33 dB vs. −5.31 dB, *p* = 0.001).

**Conclusions:**

This study indicates a considerable proportion of atypical IIH cases among IIH + TSS patients with conventional medical treatment failure. Moreover, VSS seems to be an effective and safe therapeutic modality for atypical IIH + TSS.

## Graphical abstract

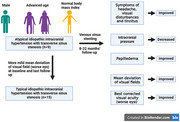

## 1 Introduction

Idiopathic intracranial hypertension (IIH) is characterized by elevated intracranial pressure (ICP) of unknown etiology. The major symptoms are headache, tinnitus, and visual disturbance. The incidence of IIH ranges between 0.5 and 2 per 100,000 individuals in the general population (Raoof and Hoffmann 2021). Without appropriate treatment, approximately 25% of patients may suffer from permanent visual impairment (Colman et al. 2024). The frequent coexistence of venous sinus stenosis in IIH highlights the importance of venous sinus stenting (VSS) therapy as a critical intervention strategy (Zhao et al. 2022; Kabanovski et al. 2022), while transverse sinus stenosis (TSS) is the most common type of venous sinus stenosis. IIH predominantly affects young individuals, particularly women of childbearing age. However, there are atypical IIHs, with normal body mass index (BMI), advanced age, and male sex (Donaldson et al. 2022; Bruce et al. 2009, 2010, 2009, 2010). The clinical profiles and stenting outcomes of atypical IIH patients might differ from those of typical patients, as atypical patients might have a different pathophysiological mechanism underlying IIH compared with typical patients. In the present study, IIH + TSS patients who were refractory, intolerant, or unwilling to receive conventional medical treatments and received VSS treatments were retrospectively reviewed in a prospectively maintained IIH + VSS database. The clinical profiles and stenting outcomes of patients were compared between typical and atypical patients and from baseline to the last follow‐up visit. This pilot study preliminarily represents the proportion, clinical profile, and stenting outcome of atypical IIH + TSS patients who fail conventional medical treatments.

## 2 Method

### 2.1 Study Subjects

IIH + TSS patients who were refractory, intolerant, or unwilling to receive conventional medical treatments and received VSS therapy were retrospectively reviewed in a prospectively recruited database, which recorded IIH patients’ comorbid with venous sinus stenosis at Beijing Tiantan Hospital from May 2021 to December 2022. Patients included in the study had a definite IIH diagnosis according to modified Friedman criteria (Appendix S1) (Friedman et al. 2013), a pre‐stenting transstenotic gradient (TSG) ≥ 8 mmHg (Fargen et al. 2018), and at least 6 months of follow‐up. The study followed the Helsinki Declaration, and written informed consent was obtained from all participants. The research plan was approved by the ethical committee of Beijing Tiantan Hospital (KY2016‐039‐02).

### 2.2 Conventional Medical Treatment Procedures

The conventional medical treatments were performed at our center as previously reported (Raynald et al. 2021) and included the following: the prescription of acetazolamide (0.25–1.5 g/day), a weight loss program, short‐term mannitol (0.25–1 g/kg of body weight per day), repeated therapeutic lumbar puncture (LP) to remove cerebrospinal fluid (20 mL per time), and the use of analgesics for headache. Patients who were refractory to the treatment were defined as unchanged/worsened/relapsed self‐reported symptoms/objective ophthalmic examinations after maximum use of conventional treatment methods. Patients who were unwilling to receive conventional treatments were defined as those who had improved self‐reported symptoms/objective ophthalmic examinations after conventional medical treatments but still desired further endovascular intervention. Patients who were intolerant to treatment were defined as intolerance to conventional treatment methods, such as electrolyte disturbances caused by acetazolamide or unbearable discomfort during the weight loss process. The decision on endovascular intervention was reached through consensus among the neurointerventionalist, neurologist, ophthalmologist, and patient.

### 2.3 Definition of Atypical IIH

Atypical IIH was defined according to prior studies (Donaldson et al. 2022; Bruce et al. 2009, 2010, 2009, 2010; Zhou 2002; Mollan et al. 2018). Patients with normal BMI, advanced age, and male sex were considered to have atypical IIH. However, there is no consensus cut‐off value for BMI and age. Bruce et al. (2010) used 50 years of age as the cut‐off value for advanced‐stage of atypical IIH. They employed a data‐driven method to establish this cut‐off value, which is approximately the mean plus two standard deviations of age in their cohort. Donaldson et al. (2022) selected 40 as the threshold for categorizing individuals as being of advanced age. In our cohort, we chose the integer closest to the 95th percentile of age (51.5 years old) as the cut‐off value (50 years old). Moreover, our cut‐off value is close to the natural menopausal age (48.7 years old) in China (Wang et al. 2021). As IIH typically occurs in women of childbearing age, we believe that the age after natural menopause is atypical. For BMI, Mollan et al. (2018) used 30 kg/m^2^ as the cut‐off value, Donaldson et al. (2022) used 26 kg/m^2^ as the cut‐off value, and Bruce et al. (2010) used 25 kg/m^2^ according to the World Health Organization BMI guidelines. In the present study, the patients included all belonged to the Han Chinese ethnic group, and according to the Cooperative Meta‐Analysis Group of the Working Group on Obesity in China, a normal BMI was defined as < 24 kg/m^2^ for the Chinese population (Zhou 2002). Thus, we used 24 kg/m^2^ as the cut‐off value.

### 2.4 Intervention Procedures

Initially, under local anesthesia, anteroposterior, lateral, and oblique venous sinus images were obtained via digital subtraction angiography (DSA) to confirm the existence of TSS. Subsequently, venous sinus manometry was performed with a Rebar‐027 microcatheter (Medtronic, USA) guided by a HI‐TORQUE Command 0.014 in. 300 cm guidewire (Abbott, USA) to the superior sagittal sinus (SSS) through femoral vein access. An Avance CS^2^ anesthesia workstation (Datex‐Ohmeda, USA) was connected to Rebar‐27 through a disposable DELTRANII pressure transducer (Utah Medical Products, USA). Manometry was performed at the SSS, torcula, transverse sinus, sigmoid sinus, internal jugular vein just below the bulb, superior vena cava‐atrial junction, and proximal and distal ends of the TSS after zero setting at the midaxillary line (Fargen et al. 2020).

Next, following the administration of general anesthesia, access to the targeted TSS lesion was established via a Mach1 guide catheter (Boston Scientific, USA), a Radifocus guidewire (Terumo Corporation, Japan), a 205 cm long, 0.014‐in. Transcend microwire (Boston Scientific, USA), and a balloon or self‐expanding Wallstent/Precise carotid stent catheter (Boston Scientific or Cordis, USA), optionally in conjunction with a Navien intracranial support catheter (Medtronic, USA). Under fluoroscopic guidance, the stent was precisely deployed at the target stenosis site. Pre‐stenting or post‐stenting dilation was performed according to the judgment of the interventionalist. A repeat manometry was performed under general anesthesia after the stenting procedure. During the procedure, intravenous heparin was administered to elevate the activated clotting time to more than 250 sec (Albuquerque et al. 2011).

Prior to the stenting procedure, patients were initiated on a regimen comprising 100 mg of aspirin and 75 mg of clopidogrel (for at least 3 days). Following stent implantation, a standardized 3‐month regimen of dual antiplatelet therapy was prescribed, subsequently transitioning to a single antiplatelet agent for an additional 3–9 months. Postprocedurally, routine recommendations did not encompass weight reduction therapy, the prescription of acetazolamide, the administration of mannitol, or therapeutic LP for patients.

### 2.5 Data Collection

The following baseline parameters were recorded: patient age, sex, BMI, and duration of the disease from symptom onset to admission. Furthermore, symptoms and signs, including headache, visual disturbances, papilledema, and tinnitus, were documented. The symptoms of headache encompassed various types, namely pressure‐like, throbbing, tension‐type‐like, migraine‐like, and nonspecific headache (Mollan et al. 2021). Visual disturbances included transient visual obscurations, blurred vision, diplopia, and visual field impairments (Raynald et al. 2023). ICP was measured by LP in the lateral decubitus position, with at least 3 days' discontinuation of any ICP‐lowering medications. During the interventional procedure, the pre‐stenting and post‐stenting TSGs were measured, and any periprocedural complications were recorded. At the baseline and follow‐up visits, visual acuity (as evaluated via a Snellen vision chart), degree of papilledema (as evaluated via fundus photography), and visual field tests (as evaluated via the Humphrey Field Analyzer SITA Faster program 24‐2) were performed. The severity of papilledema was graded according to the Frisen scale (Frisén 1982) and prospectively recorded in our database, while the best corrected visual acuity (BCVA) in the logarithm of the minimum angle of resolution (logMAR), as well as the mean deviation (MD) of the visual field in decibels (dB), were retrospectively collected and recorded.

Two senior neurointerventionalists, D.M and X.T, analyzed the DSA images. The development of venous sinuses can be categorized into three classes: left‐dominance or right‐dominance, signifying a substantial (≥ 40%) disparity in caliber between the two sinuses; and codominance, representing a bilateral balance in venous sinus caliber (Durst et al. 2016). The stenosis types, namely intrinsic and extrinsic, were differentiated based on previously published morphological characteristics (Raynald et al. 2021; Tian et al. 2021). Extrinsic stenosis reveals a lengthy, smooth, and progressive narrowing, with an absence of intraluminal obstructions. Conversely, intrinsic stenosis is distinguished by the presence of intraluminal obstructions, such as prominent arachnoid granulations or fibrous septations, forming an obvious filling defect within the lumen and typically presents with a circumscribed pattern of stenosis.

Data related to the alleviation of clinical manifestations, post‐stenting ICP (through the LP), and stent‐adjacent stenosis (through DSA) at the last follow‐up visit were collected. Symptoms and signs were evaluated and compared with those at baseline. The three main symptoms (headache, visual disturbance, tinnitus) were then categorized into three classes modified from the Higgins et al. (2003) method: (1) asymptomatic (completely resolved or normal at baseline), (2) improved (existence of self‐reported alleviated residual symptoms compared with symptom onset, which did not require further intervention) (Raynald et al. 2021), and (3) not improved (unchanged, worsened, newly developed, or relapsed). The outcome of papilledema was categorized into three classes: (1) completely resolved (both eyes had a Frisen grade of 0), (2) improved (both eyes had a lower Frisen grade than at baseline), and (3) not improved (any of the eyes had an equal or higher Frisen grade than at baseline).

### 2.6 Statistical Analysis

Statistical analysis was performed via SPSS software (version 25.0, IBM, USA). To assess the normality of continuous variables, the Kolmogorov–Smirnov test and the Shapiro–Wilk test were employed. In cases where either of these tests yielded statistical significance, the variable was deemed to exhibit a nonnormal distribution. Subsequently, for the purpose of comparing variables between two groups, different statistical tests were applied on the basis of the data distribution: the independent *t*‐test was utilized for normally distributed continuous data, the Mann–Whitney *U* test was applied for nonnormally distributed continuous data, and for categorical data, the chi‐square test or two‐tailed Fisher's exact test was employed. The paired *t*‐test (for normally distributed data) and Wilcoxon signed‐rank test (for nonnormally distributed data) were used to compare numerically continuous parameters at baseline and at the last follow‐up.

## 3 Results

### 3.1 Baseline and Periprocedural Characteristics

Data were available for 24 IIH + TSS patients. The median age was 37 years, and 95.8% were female. The median follow‐up duration was 12 months (range 8–22 months). There were 9/24 atypical patients (37.5%), 6 with a BMI < 24 kg/m^2^, one male, and two aged ≥ 50 years.

Atypical patients had a lower BMI than typical patients (23.44 kg/m^2^ vs. 28.65 kg/m^2^, *p* < 0.001). The median disease duration before treatment was 8 months, the median ICP was 300 mmH_2_O, and the median pre‐stenting TSG was 13 mmHg. Headache, visual disturbances, papilledema, and tinnitus occurred in 66.7%, 77.8%, 100.0%, and 44.4% of atypical patients, respectively, and 55.6% of atypical patients had extrinsic VSS, with a median ICP of 300 mmH_2_O and a median pre‐stenting TSG of 13 mmHg. Stents with 7–9 mm in length and 40–50 mm in diameter were deployed at the site of TSS. After the procedure, the instant TSG effectively decreased to a median of 0 mmHg (in the quartile range of 0–1 mmHg). Atypical patients had more mild visual field defects compared with typical patients, as reflected by the MD of the worse eye (median of −7.63 dB in typical patients and median of −3.08 dB in atypical patients, *p* = 0.027). Compared with those of typical patients, the baseline and periprocedural characteristics of atypical patients did not significantly differ, except for the baseline BMI and MD of the worse eye (Table 1). During the procedure, no periprocedural complications, such as subdural hematoma, stent migration, in‐stent thrombosis, femoral pseudoaneurysm, contrast agent allergic dermatitis, or failure of stent delivery/deployment, were observed.

**TABLE 1:** Baseline and periprocedural characteristics of IIH + TSS patients.

	Typical (*N* = 15)		Atypical (*N* = 9)		*p*
	Number/median	Range/proportion	Number/median	Range/proportion	
Age (years)	33	16–48	38	25–52	0.522
Sex					0.375
Male	0	0.0%	1	11.1%	
Female	15	100.0%	8	88.9%	
BMI (kg/m^2^)	28.65	25.00–40.23	23.44	20.32–28.24	**< 0.001**
Response to conventional medical treatment					0.665
Unwilling	5	33.3%	4	44.4%	
Intolerant	1	6.7%	1	11.1%	
Refractory	9	60.0%	4	44.4%	
Time since symptom onset (months)	5	0.67–24	8	1–24	0.238
Follow‐up duration (months)	12	10–21	12	8–22	0.953
Symptoms and signs					
Headache	7	46.7%	6	66.7%	0.423
Visual disturbances	15	100.0%	7	77.8%	0.130
Blurred vision	14	93.3%	6	66.7%	0.130
Visual field impairment	2	13.3%	1	11.1%	0.692
Transient visual obscurations	2	13.3%	3	33.3%	0.255
Diplopia^a^	2	13.3%	1	11.1%	0.692
Papilledema	15	100.0%	9	100.0%	—
Frisen grade					
Worse eye	3	1–4	3	2–4	0.815
Fellow eye	3	1–4	3	2–3	> 0.999
Tinnitus	2	13.3%	4	44.4%	0.150
BCVA (logMAR)					
Worse eye	0.097	−0.079 to 2.602	0.097	−0.079 to 0.824	0.347
Fellow eye	0.000	−0.176 to 2.602	0.097	−0.176 to 0.301	0.815
MD (dB)^b^					
Worse eye	−7.63	−35.00 to −1.52	−3.08	−4.70 to 0.55	**0.027**
Fellow eye	−2.87	−32.64 to 0.76	−2.37	−3.39 to 0.00	0.246
Sinus development					0.513
Left‐dominance	3	20.0%	1	11.1%	
Right‐dominance	9	60.0%	4	44.4%	
Codominance	3	20.0%	4	44.4%	
Stenosis type					0.099
Intrinsic	12	80.0%	4	44.4%	
Extrinsic	3	20.0%	5	55.6%	
ICP (mmH_2_O)	330	250–330	300	250–330	0.064
Pre‐stenting TSG (mm Hg)	16	8–43	13	8–20.5	0.599
Number of stents	1	1–2	1	1–1	0.640
Stent1					0.672
7 mm × 40 mm	6	40.0%	2	22.2%	
7 mm × 50 mm	4	26.7%	2	22.2%	
8 mm × 40 mm	5	33.3%	4	44.4%	
9 mm × 40 mm	0	0.0%	1	11.1%	
Stent2					> 0.999
None	13	86.7%	9	100.0%	
7 mm × 40 mm	1	6.7%	0	0.0%	
7 mm × 50 mm	1	6.7%	0	0.0%	
Post‐stenting TSG (mm Hg)	1	0–3	0	0–1	0.599
Complication					—
Subdural hematoma	0	0.0%	0	0.0%	
Stent migration	0	0.0%	0	0.0%	
In‐stent thrombosis	0	0.0%	0	0.0%	
Femoral psudoanurysm	0	0.0%	0	0.0%	
Contrast agent allergic dermatitis	0	0.0%	0	0.0%	
Failure of stent delivery/deployment	0	0.0%	0	0.0%	

Abbreviations: BCVA, best corrected visual acuity; BMI, body mass index; dB, decibel; ICP, intracranial pressure; IIH, idiopathic intracranial hypertension; logMAR, logarithm of the minimum angle of resolution; MD, mean deviation; TSG, transstenotic gradient; TSS, transverse sinus stenosis.

^a^ No patient had the symptom of diplopia independent of other symptoms of visual disturbance (blurred vision, visual field impairment, or transient visual obscurations).

^b^ The MD values of six patients were missing.

The bold values means the P values are < 0.05 (statistically significant).

### 3.2 Outcomes of Stenting in IIH + TSS Patients at the Last Follow‐Up

While the BMI at the last follow‐up was lower in atypical patients than in typical patients (23.05 kg/m^2^ vs. 28.89 kg/m^2^, *p* = 0.016), the percentage of body weight change between groups did not have a statistical difference (−1.56% vs. −3.27%, *p* = 0.290). The median ICP at the last follow‐up was 180 mmH_2_O in the atypical IIH group. No atypical IIH patients had elevated ICP (> 250 mmH_2_O) or severe stent‐adjacent stenosis (> 50%) at the last follow‐up, but 2/15 (13.3%, one intrinsic stenosis and one extrinsic stenosis at baseline) of the typical IIH patients had stent‐adjacent stenosis (> 50%). However, the proportion of patients with stent‐adjacent stenosis did not significantly differ between typical and atypical patients. Headache, visual disturbance, papilledema, and tinnitus were asymptomatic at follow‐up in 88.9%, 66.7%, 100.0%, and 100.0% of the atypical IIH patients, respectively. The percentages of body weight change, ICP, stent‐adjacent stenosis, and symptom improvement in typical and atypical IIH patients were comparable (Table 2). With respect to objective visual outcomes, the degree of papilledema improvement, BCVA of both eyes, and MD of the fellow eye were comparable between the groups; however, the MD of the worse eye in the atypical group was greater than that in the typical group (−1.33 dB vs. −5.31 dB, *p* = 0.001).

**TABLE 2:** Clinical outcomes of stent‐treated IIH + TSS patients.

	Typical (*N* = 15)		Atypical (*N* = 9)		
	Number/median	Range/proportion	Number/median	Range/proportion	*p*
Follow‐up BMI (kg/m^2^)	28.89	19.13–38.67	23.05	20.20–29.73	**0.016**
Body weight change (%)^a^	−3.27	−27.03 to 3.51	−1.56	−4.96 to 15.38	0.290
ICP (mmH_2_O)	190	120–280	180	100–210	0.175
ICP > 250 mmH_2_O	2	13.3%	0	0.0%	0.511
Stent‐adjacent stenosis (> 50%)	2	13.3%	0	0.0%	0.511
Headache					> 0.999
Asymptomatic	13	86.7%	8	88.9%	
Improved	1	6.7%	1	11.1%	
Not improved	1	6.7%	0	0.0%	
Visual disturbance					0.588
Asymptomatic	11	73.3%	6	66.7%	
Improved	4	26.7%	2	22.2%	
Not improved	0	0.0%	1	11.1%	
Papilledema					0.511
Completely resolved	13	86.7%	9	100.0%	
Improved	2	13.3%	0	0.0%	
Not improved	0	0.0%	0	0.0%	
Tinnitus					> 0.999
Asymptomatic	14	93.3%	9	100.0%	
Improved	0	0.0%	0	0.0%	
Not improved	1	6.7%	0	0.0%	
BCVA (logMAR)					
Worse eye	0.000	−0.079 to 1.301	0.000	0.000–0.523	0.347
Fellow eye	0.000	−0.079 to 2.602	0.000	0.000–0.222	0.861
MD (dB)^b^					
Worse eye	−5.31	−32.43 to −0.32	−1.33	−3.03 to 0.62	**0.001**
Fellow eye	−1.40	−31.05 to 1.48	−0.28	−3.28 to 1.07	0.328

Abbreviations: BCVA, best corrected visual acuity; BMI, body mass index; dB, decibel; ICP, intracranial pressure; IIH, idiopathic intracranial hypertension.; logMAR, logarithm of the minimum angle of resolution; MD, mean deviation; TSS, transverse sinus stenosis.

^a^ The body weight change was calculated as the percentage difference between the body weight at the last visit and at baseline. It was determined by (last visit body weight—baseline body weight)/baseline body weight × 100%.

^b^ Six patients’ visual field results were missing.

The bold values means the P values are < 0.05 (statistically significant).

In typical and atypical patients, ophthalmic examinations (BCVA, MD, and Frisen grade) of both eyes, BMI, and ICP between baseline and the last visit were compared (Table 3). Typical patients had decreased BMI (the data were normally distributed, with a mean BMI of 30.08 kg/m2 at baseline and 28.83 kg/m^2^ at follow‐up, *p* = 0.042); however, atypical patients did not have a decreased BMI during follow‐up (the data were normally distributed, with a mean BMI of 23.59 kg/m^2^ at baseline and 23.80 kg/m^2^ at follow‐up, *p* = 0.706). The stenting intervention significantly decreased the ICP and grade of papilledema and improved the BCVA in the worse eye and MD in the fellow eye in both typical and atypical patients. The change of MD in the worse eye did not reach statistical significance (−7.63 dB at baseline and −5.31 dB at the last follow‐up, *p* = 0.050) in typical patients, while it had a statistical difference (−3.08 dB at baseline and −1.33 dB at the last follow‐up, *p* = 0.009) in atypical patients.

**TABLE 3:** Comparison of BMI, ICP, and ophthalmic examinations between baseline and the last visit in typical and atypical patients.

	Baseline		Last visit		
	Median	Range	Median	Range	*p*
Typical patients					
BMI (kg/m^2^)	28.65	25.00–40.23	28.89	19.13–38.67	**0.042**
ICP (mmH_2_O)	330	250–330	190	120–280	**0.001**
Frisen grade					
Worse eye	3	1–4	0	0–1	**0.001**
Fellow eye	3	1–4	0	0–2	**0.001**
BCVA (logMAR)					
Worse eye	0.097	−0.079 to 2.602	0.000	−0.079 to 1.301	**0.023**
Fellow eye	0.046	−0.176 to 2.602	0.000	−0.079 to 2.602	0.213
MD (dB)^a^					
Worse eye	−7.63	−35.00 to −1.52	−5.31	−32.43 to −0.32	0.050
Fellow eye	−2.87	−32.64 to 0.76	−1.40	−31.05 to 1.48	**0.010**
Atypical patients					
BMI (kg/m^2^)	23.44	20.32–28.24	23.05	20.20–29.73	0.706
ICP (mmH_2_O)	300	250–330	180	100–210	**0.008**
Frisen grade					
Worse eye	3	2–4	0	0–0	**0.007**
Fellow eye	3	2–3	0	0–0	**0.006**
BCVA (logMAR)					
Worse eye	0.097	−0.079 to 0.824	0.000	0.000–0.523	**0.046**
Fellow eye	0.097	−0.176 to 0.301	0.000	0.000–0.222	0.612
MD (dB)^b^					
Worse eye	−3.08	−4.70 to −0.55	−1.33	−3.03 to 0.62	**0.009**
Fellow eye	−2.37	−3.39 to 0.00	−0.28	−3.28 to 1.07	**0.041**

Abbreviations: BCVA, best corrected visual acuity; BMI, body mass index; dB, decibel; ICP, intracranial pressure; logMAR, logarithm of the minimum angle of resolution; MD, mean deviation.

^a^ The visual field results of four patients were missing.

^b^ The visual field results of two patients were missing.

The bold values means the P values are < 0.05 (statistically significant).

## 4 Discussion

The present study showed a considerable proportion of atypical IIH (37.5%) in IIH + TSS patients who experienced failure of conventional medical treatment, emphasizing the paramount importance of delving into atypical patients’ clinical characteristics and stenting outcomes. Notably, procedural features and stenting responses were comparable between typical and atypical IIH patients. These findings suggest that stent therapy is a feasible and efficacious therapeutic modality for patients with atypical IIH.

Despite the use of different age and BMI cut‐off values, Donaldson et al. (2022) reported comparable visual outcomes between atypical and typical IIH patients, which is consistent with our study. However, Donaldson et al. (2022) retrospectively studied general IIH patients, not IIH patients with conventional medical treatment failure, as in our study. Interestingly, Donaldson et al. (2022) reported that in atypical and typical IIH patients, the number of patients requiring surgical treatment was comparable. Donaldson et al. (2022) also reported that baseline clinical manifestations might be different between IIH subtypes, as older patients less frequently reported headaches. The study reported 42.9% atypical IIH in the general IIH population, similar to our data (37.5%). Cho et al. (2024) also reported a high proportion of atypical IIH (16.9% were male, while 54.2% had a BMI < 30 kg/m^2^) in the Asian population through a multicenter prospective study conducted in Korea.

Bruce et al. (2010) reported that in atypical IIH patients, older patients tended to report headaches less frequently and visual changes more frequently. Furthermore, compared with typical patients, older patients had a lower chance of receiving medication and a greater chance of suffering from persistent papilledema but similar visual outcomes in aspects of the visual field and visual acuity. The initial symptoms and treatment outcomes between normal‐BMI patients and high‐BMI patients were comparable in Bruce et al. (2010)’s study. The study reported a 4% proportion of individuals with a normal BMI and a 5% proportion of elderly IIH patients.

Bruce et al. (2009) reported in a separate study that male IIH patients exhibited a lower frequency of headache complaints and a greater frequency of visual disturbances. Visual field and visual acuity outcomes were worse in men than in women. This study reported 9% of the men in the general IIH population, which was higher than the 4.2% reported in our study.

Raper et al. (2018) reported the stenting outcomes of IIH patients with normal or high BMIs in a retrospective cohort. They found that BMI was positively correlated with the pre‐stenting TSG but was not correlated with the post‐stenting TSG. However, IIH patients with normal or high BMIs had similar symptomatic improvements and stent‐adjacent stenosis rates after VSS.

Other evidence also indicates that demographic features are associated with the clinical manifestations or outcomes of IIH. A long‐term observation of 39 stented IIH patients over 17 years revealed that female sex was associated with restenosis (Kumpe et al. 2017). Weight gain is associated with poor visual outcomes (Chagot et al. 2017), whereas weight loss is associated with disease remission in IIH patients (Abbott et al. 2023; Gafoor et al. 2017). Age was negatively correlated with the ICP (Gafoor et al. 2017), and IIH patients > 50 years of age had a lower ICP compared with younger patients (Downie et al. 2021).

As IIH typically occurs in obese females of childbearing age, the pathophysiology of typical IIH might be associated with hormone dysregulation, obesity‐related inflammation, and metabolomic alterations (Colman et al. 2024; Alimajstorovic et al. 2023). However, in IIH patients with atypical demographic characteristics, other pathophysiological pathways might function. The potential disparity in pathogenesis between typical and atypical IIH necessitates the observation of more cases to clarify the distinct clinical profiles and therapeutic effects of stenting in patients with various subtypes of IIH.

This study has several limitations. First, as a pilot study, the small sample size limits the statistical power to compare clinical baseline characteristics and stent efficacy between typical and atypical patients. Moreover, the potential heterogeneity in clinical manifestations and outcomes among normal BMI, elderly, and male atypical IIH patients may have been obscured by the small sample number of subgroups in the present study (only two elderly atypical IIH patients and one male atypical IIH patient). Thus, we did not perform subgroup analysis to achieve a more nuanced understanding of the disease. Second, the current median follow‐up duration was 12 months. To gain a more comprehensive understanding of long‐term outcomes, future studies with extended follow‐up periods are recommended. Third, the data of visual field tests and visual acuity measurements were retrospectively collected. Among these data, there were 6 MD missing values, which may have introduced bias in our analysis. Last, our database does not include information on the prescription and frequency of analgesics or preventatives for headaches, thereby limiting our ability to conduct a more objective evaluation of headache changes within our cohort.

## 5 Conclusion

This study retrospectively reviewed a prospectively maintained IIH + TSS cohort and revealed a considerable proportion of atypical IIH cases among IIH + TSS patients who experienced failed conventional medical treatments. VSS has emerged as an effective and safe therapeutic modality for atypical IIH + TSS. However, owing to the small sample size of the study and its observational nature, the conclusions should be validated by meta‐analyses and randomized controlled trials in the future.

## Author Contributions

**Shuang Song**: investigation, writing – original draft, writing – review and editing, formal analysis. **Zhongao Guan**: investigation, writing – review and editing. **Shuran Wang**: investigation, formal analysis. **Yawen Gan**: investigation, writing – review and editing. **Fangguang Chen**: investigation, writing – review and editing. **Jie He**: investigation, writing – review and editing. **Ketao Tu**: investigation, writing – review and editing. **Zhongrong Miao**: investigation, writing – review and editing. **Xu Tong**: conceptualization, investigation, writing – review and editing, funding acquisition. **Dapeng Mo**: conceptualization, investigation, funding acquisition, writing – review and editing. **Ke Pang**: conceptualization, investigation, funding acquisition, writing – review and editing.

## Ethics Statement

We followed the Helsinki Declaration and obtained written consent from all participants. The research plan was approved by the ethical committee of Beijing Tiantan Hospital (KY2016‐039‐02).

## Consent

The authors have nothing to report.

## Conflicts of Interest

The authors declare no conflicts of interest.

### Peer Review

The peer review history for this article is available at https://publons.com/publon/10.1002/brb3.70351

## Supporting information



Supplementary Materials.

## Data Availability

The data supporting the conclusions of this article are upon reasonable request to the corresponding authors.
