# Supplementary material for: Outcomes of Stenting in Atypical Idiopathic Intracranial Hypertension Patients With Transverse Sinus Stenosis
Source: Brain Behav. 2025 Feb 19;15(2):e70351. doi: 10.1002/brb3.70351 (PMC11839763; doi:10.1002/brb3.70351)
Supplement: Supplementary file 1 — Supplementary Materials. [file BRB3-15-e70351-s001.docx]

**Supplementary file Appendix S1 Diagnostic procedure**

First, based on the 2013 modified Friedman criteria for the pseudotumor cerebri syndrome in adults, a definite pseudotumor cerebri syndrome should be diagnosed^1^. Patients should fulfill the following criteria A–E: A. papilledema; B. normal neurologic examination except for cranial nerve abnormalities; C. neuroimaging: normal brain parenchyma without evidence of hydrocephalus, mass, or structural lesion and no abnormal meningeal enhancement on magnetic resonance imaging (MRI), with and without gadolinium, for typical patients (female and obese), and MRI, with and without gadolinium, and magnetic resonance venography for others; if MRI is unavailable or contraindicated, contrast-enhanced computed tomography (CT) may be used; D. normal cerebrospinal fluid (CSF) composition; E. elevated lumbar puncture opening pressure (≥250 mm H_2_O) in a properly performed lumbar puncture. The ophthalmological examination was conducted prior to lumbar puncture to avoid any potential temporary improvement in papilloedema after the procedure.

Second, according to past medical history, family history, blood tests (at least complete blood count, blood biochemistry test, erythrocyte sedimentation rate, rheumatoid factor test, C-reactive protein test, hormone level measurement, coagulation function test, autoantibody test, and antibody screening for infectious pathogens), cardiac assessment (electrocardiogram and echocardiography), magnetic resonance venography and digital subtraction angiography, patients with any signs indicating the possibility of secondary pseudotumor cerebri were excluded, including cerebral venous abnormalities (cerebral venous sinus thrombosis; bilateral jugular vein thrombosis or surgical ligation; middle ear or mastoid infection; increased right heart pressure; superior vena cava syndrome; arteriovenous fistulas; decreased CSF absorption from previous intracranial infection or subarachnoid hemorrhage; hypercoagulable states), specific medications and exposures (antibiotics, including tetracycline, minocycline, doxycycline, nalidixic acid, and sulfa drugs; vitamin A and retinoids; hormones, including human growth hormone, leuprorelin acetate, levonorgestrel, and anabolic steroids; withdrawal from chronic corticosteroids; lithium; chlordecone), and specific medical conditions (endocrine disorders, including addison disease and hypoparathyroidism; hypercapnia, such as sleep apnea and Pickwickian syndrome; anemia; renal failure; Turner syndrome; Down syndrome).

Thus, a diagnosis of primary pseudotumor cerebri syndrome (idiopathic intracranial hypertension) was established.

**Reference**

1. Friedman DI, Liu GT, Digre KB. Revised diagnostic criteria for the pseudotumor cerebri syndrome in adults and children. *Neurology*. 2013;81(13):1159-1165. doi:10.1212/WNL.0b013e3182a55f17
